# The Human Disharmony Loop: A Case Series Proposing the Unique Role of the Pectoralis Minor in a Unifying Syndrome of Chronic Pain, Neuropathy, and Weakness

**DOI:** 10.3390/jcm14051769

**Published:** 2025-03-06

**Authors:** Ketan Sharma, James M. Friedman

**Affiliations:** 1 St Luke’s Plastic and Reconstructive Surgery, Meridian, ID 83642, USA; 2 Alpine Orthopaedic, Stockton, CA 94158, USA; james.friedman46@gmail.com

**Keywords:** scapula dyskinesia, pectoralis minor, pain, neuropathy, weakness, impingement, shoulder

## Abstract

**Background/Objectives**: Many patients evaluated by shoulder specialists suffer from intractable pain, neuropathy, and weakness. The pectoralis minor (PM) remains the only scapula muscle to receive lower trunk (C8-T1) input. We propose a novel syndrome: the Human Disharmony Loop. This model portrays how this unique PM innervation causes scapular dyskinesia, which deranges the anatomy of the upper limb girdle and produces a refractory symptom complex of pain, neuropathy, and weakness. We hypothesize that certain patients with historically intractable symptoms of the upper limb girdle may benefit from PM tenotomy. **Methods**: Ten patients of diverse etiologies presented with a similar constellation of complaints. The patients included a female athlete, a female with macromastia, a male bodybuilder, and patients with post-radiation breast cancer, post-operative shoulder arthroplasty, interscalene block injury, cervical spine disease, persistent impingement after rotator cuff repair, direct traction injury, and occupational disorder. All patients exhibited coracoid tenderness, scapula protraction with internal rotation and anterior tilt, and pain involving the neck, shoulder, and upper back. The patients demonstrated varying degrees of arm neuropathy, subacromial impingement, and occipital headaches. The patients failed all prior treatments by multiple subspecialists, including surgery. Each patient underwent isolated open PM tenotomy. **Results**: In all ten patients, PM tenotomy substantially reduced shoulder, upper back, and neck pain, cleared concomitant neuropathy, restored full motion, and eradicated occipital headaches. The response to surgery was rapid, dramatic, and durable. **Conclusions**: The unique asymmetric neurologic innervation to the sole ventral stabilizer of the scapula, the pectoralis minor, predisposes the human shoulder to neurologic and musculoskeletal imbalance. This produces the Human Disharmony Loop: a clinical syndrome spanning from the neck to the fingertips, with chronic pain, neuropathy, and weakness. These challenging patients may benefit dramatically from isolated PM tenotomy.

## 1. Introduction

“The central theme of the shoulder is motion” [1]. The shoulder’s full arc of motion encompasses overhead, across-body, and behind-body reach, and it involves eighteen muscles acting across four articulations, chiefly the glenohumeral and scapulothoracic. The scapula dynamically links the body with the arm, coordinating both mobility and stability. Importantly, the scapula must glide along the thoracic wall to perform its two critical functions. First, the scapula maintains optimal length–tension relationships for the muscles acting on the glenohumeral joint, so they can provide effective excursion despite shoulder position. Second, the scapula prevents impingement of the rotator cuff between the acromion and the humerus. The peri-scapular chain, comprising the trapezius, rhomboids, levator scapulae, serratus anterior, and the pectoralis minor (PM), coordinates this motion. Of these, the trapezius and serratus anterior are the principal rotators of the scapula during this scapulothoracic rhythm, while the PM acts to stabilize the scapula against the thorax. Unopposed pull of the PM leads the scapula to move in a direction of protraction (lateral translation) with anterior tilt and internal rotation. Full pain-free motion truly relies on a harmony of musculoskeletal and neurologic forces.

Despite noteworthy advances in arthroscopic and open techniques, implant design, and understanding of biomechanics, many patients continue to suffer from chronic pain, neuropathy, and weakness. These patients notoriously fail to improve despite treatment by therapists, pain management physicians, chiropractors, neurologists, and spine, shoulder, and sports surgeons. They are often tagged with challenging and vague diagnoses including thoracic outlet syndrome (TOS), scapular dyskinesia, myofascial tightness, fibromyalgia, complex regional pain syndrome (CRPS), occupational shoulder disorder (ORD), or work-related musculoskeletal disorder (WRMD). These historically challenging and intractable scenarios frustrate surgeons, therapists, and patients alike.

In this paper, we report a case series of ten patients who presented with a similar constellation of refractory pain, neuropathy, and weakness. Each patient exhibited medial coracoid tenderness, neck, shoulder, and upper back pain and a specific scapula malposition of protraction and internal rotation with anterior tilt. In addition, patients demonstrated varying degrees of neuropathy at the thoracic outlet, suprascapular notch, quadrilateral space, and/or radial and carpal tunnel; rotator cuff impingement (limited ROM); and occipital headaches. Patients stemmed from diverse and non-overlapping etiologies, ranging from female athletes to breast cancer survivors to post-operative arthroplasty patients. Each failed all previous treatments. Crucially, each patient responded to an isolated tenotomy of the PM off the coracoid process, producing durable improvement of all symptoms, including reduction of refractory pain, clearance of concomitant neuropathy, elimination of headaches, and restoration of ROM. Based on our experience, we propose a novel unifying clinical syndrome that (1) explains the similar presentation, (2) accounts for the diverse population, (3) elucidates the dramatic response to isolated PM tenotomy, and (4) unites these considerations with a known understanding of biomechanics and pathology. We hypothesize that the isolated lower trunk neurologic innervation to the PM underlies the drastic improvement in the symptoms seen.

## 2. Materials and Methods

**Figure 1:**
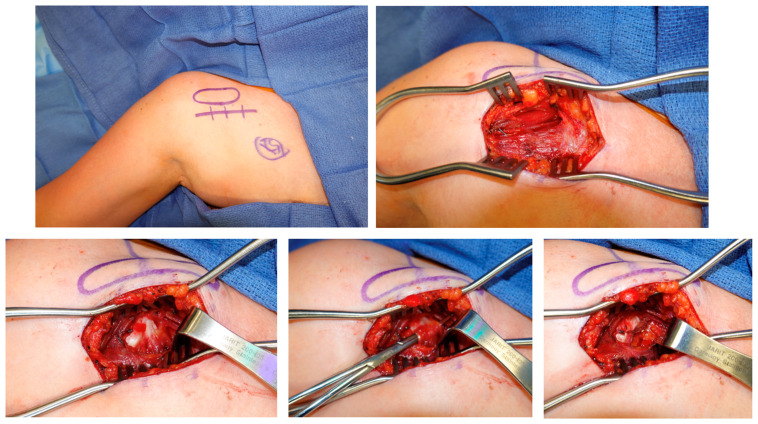
Technique for Pectoralis Minor Tenotomy. Open technique. (**Top left**) ~4 cm transverse incision inferior to palpable coracoid process. (**Top right**) Dissection down to deltoid (up), pectoralis major (down), and exposed cephalic vein (center). (**Bottom left**) Further dissection down to coracoid with conjoined tendon (left) and pectoralis minor tendon (right). (**Bottom center**) Isolating the pec minor tendon for release. (**Bottom right**) Complete pectoralis minor release off coracoid.

This is a prospective case series of patients presenting to two clinics. All were treated by a fellowship-trained board-certified hand or sports surgeon. The inclusion criteria included: age >18 years, and physical exam with medial coracoid tenderness (at the PM insertion) and scapula malposition of protraction with anterior tilt and internal rotation. The exclusion criteria included: follow-up <6 months. Patients were evaluated pre-operatively and then at 2, 6, 12, and 24 weeks. Patients completed self-reported pain diagrams and symptom questionnaires. A certified hand and physical therapist (PT) independently performed a full brachial plexus and musculoskeletal evaluation. Provocative testing identified neuropathic lesions at the thoracic outlet, PM, suprascapular notch, quadrilateral space, radial and carpal tunnels. Musculoskeletal exams included Medical Research Council (MRC) grade muscle testing and shoulder range of motion (ROM) values in forward flexion, abduction, and external rotation planes. Each patient underwent isolated PM tenotomy through an open direct approach (Figure 1). The outcomes included pain scores, presence of headaches, presence of neuropathic lesions, and shoulder ROM. Institutional Review Board (IRB) approval was obtained and need for consent was waived by the ethics committee as all data were anonymous, the study was observational only and involved standard of care treatments, and the study posed minimal risk to the included patients. This study follows the CARE guidelines.

## 3. Results

**Figure 2:**
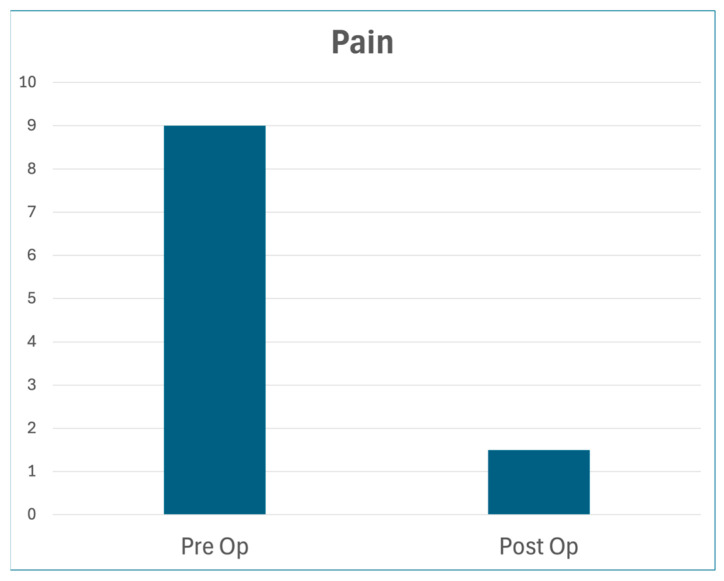
Self-Reported Pain Scores. Median pain score (out of 10) before and after surgery, with a minimum of 6 months follow-up.

Ten patients originated from diverse etiologies (Table 1) but presented with similar signs and symptoms (Table 2). The median age was 42. Each had failed all previous treatments by multiple subspecialists. In addition to the variable symptoms listed below, each patient exhibited medial coracoid tenderness with specific scapular dyskinesia of protraction, internal rotation, and anterior tilt, and endorsed shoulder pain extending to the neck and upper back. Median pre-op pain was 9.0/10; median post-op pain was 1.5/10. All patients had a minimum of 6 months follow-up (Figure 2).

Case Examples

Patient 1. The 56 y/o RHD female presented with a history of left breast cancer s/p-mastectomy with adjuvant radiation. Since then, she suffered 8/10 left arm pain from the shoulder to the dorsal forearm, along with hand numbness and weakness. She trialed physical therapy and pain management for >6 years with no relief. She underwent a left PM tenotomy. At 2 weeks post-operatively, all symptoms had resolved except for mild surgical incision pain, which remained durable at the most recent follow-up.

Patient 2. The 31 y/o RHD female with a history of macromastia presented with years of right shoulder and neck pain, forearm burning, hand numbness/tingling, and severe ipsilateral occipital migraines. She had been treated with physical therapy, chiropractors, pain management, and neurology, with minimal relief. On exam, she was also provocative at the carpal tunnel and quadrilateral space. She underwent isolated right PM tenotomy, resulting in resolution of all symptoms including migraines, shoulder pain, and other neuropathic lesions.

**Figure 3:**
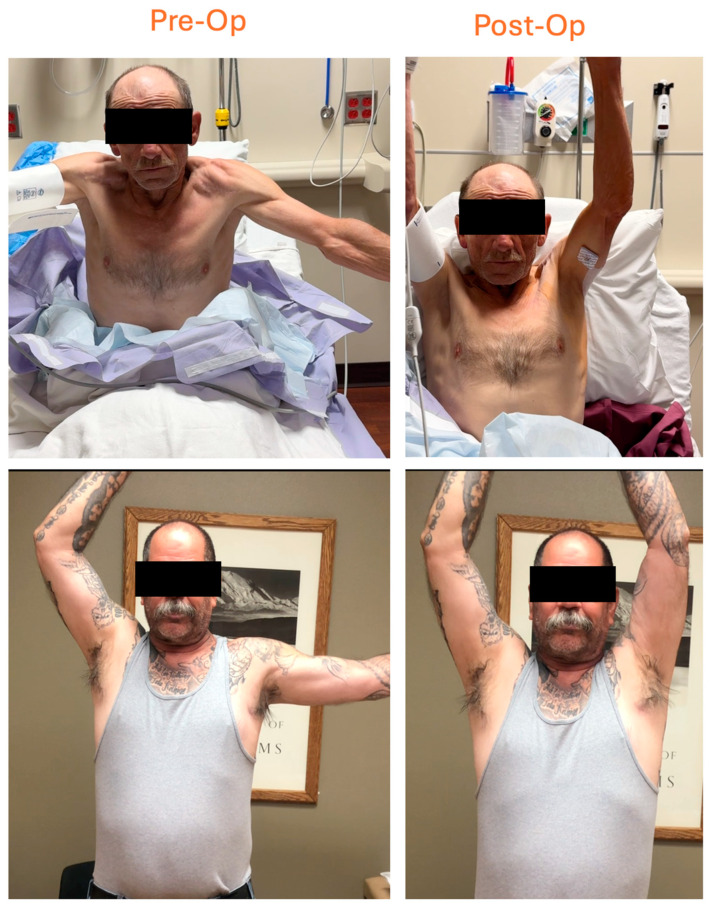
Complete Restoration of Range of Motion. Persistent weakness and limited ROM following reverse total shoulder arthroplasty (**top left**) and despite arthroscopic repair of leading-edge supraspinatus tear (**bottom left**). Both responded dramatically to surgery restoring full motion (**right**).

Patient 3. The 63 y/o RHD male with a history of left reverse TSA 16 months prior presented with continued shoulder pain, constant hand numbness/tingling, and principal complaint of shoulder weakness. He exhibited active abduction and external rotation ROM to 60 degrees only; he had a positive scratch-collapse test at the quadrilateral space, suprascapular notch, and distal radial and carpal tunnels. After left PM tenotomy, he immediately noted full shoulder strength with 180 degrees of abduction. Additionally, all other neuropathic lesions resolved (Figure 3).

Patient 4. The 59 y/o RHD male bodybuilder with no history of trauma presented with progressive multi-year history of severe 10/10 left shoulder, neck, and upper back pain. He had undergone four prior surgeries (arthroscopy with biceps tenodesis, revision tenodesis and labral repair, arthroscopic debridement ×2) and physical therapy, with no relief. After isolated PM tenotomy, he exhibited complete resolution of all symptoms.

Patient 5. The 21 y/o RHD female college athlete (swimming, volleyball) without a history of trauma presented with progressive right shoulder and neck pain and hand numbness and weakness. On exam, she was also provocative at the thoracic outlet. Her symptoms were severe enough to prevent playing sports. She had been unsuccessfully treated at a tertiary academic center for presumed thoracic outlet syndrome, including scalene botulinum toxin injections. Right PM tenotomy produced rapid and complete resolution of all symptoms, allowing full return to sport.

**Figure 4:**
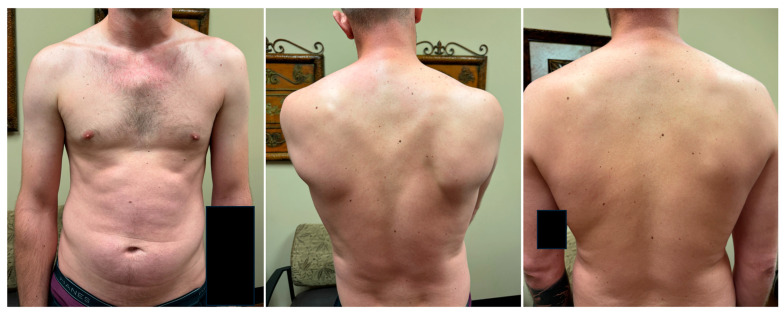
Shoulder Posture and Scapula Malposition in the Human Disharmony Loop. Pre-operatively, disharmonic right shoulder exhibits drooping and internal rotation (“hunched”) posture (**left**). Pre-operatively, disharmonic right scapula exhibits protraction, anterior tilt, and internal rotation (**middle**). Two weeks post-operatively, the shoulder and scapula are symmetric to the normal side with scapulothoracic kinesthesia restored, correlating with symptomatic improvement (**right**).

Patient 6. The 28 y/o RHD male presented 3 years after SLAP tear repair with pre-operative interscalene regional block. He exhibited unrelenting 10/10 right neck and shoulder pain with occipital migraines that precluded working. On exam, he was provocative at the quadrilateral space. EMG confirmed denervation of biceps and infraspinatus, consistent with iatrogenic regional block injury to the upper trunk. He had been previously treated by pain management, neurology, physical therapy, and shoulder and sports specialists, with no relief. He admitted to being “suicidal” due to pain. He responded very well but temporarily to a coracoid injection in clinic and subsequently underwent isolated PM tenotomy. This produced rapid improvement in all symptoms, restored normal scapular positioning (Figure 4), and cleared his quadrilateral space lesion as well. He thereby transitioned off all narcotics and returned to work.

Patient 7. The 59 y/o RHD male with a history of biceps tenodesis and revision rotator cuff repair 5 months prior presented with severe chronic and worsening left shoulder pain and ulnar hand numbness, preventing work. He had no history of trauma. On exam, he exhibited positive impingement signs and active shoulder abduction to 100 degrees. He had complete relief of symptoms with a diagnostic coracoid injection. He thereafter underwent isolated PM tenotomy. Two weeks post-operatively, his pain and impingement symptoms had resolved, he exhibited full pain-free ROM of the shoulder, he had improvement of distal neurologic symptoms, and he returned to work.

Patient 8. The 42 y/o RHD female with a history of cervical stenosis s/p- two prior cervical fusions, presented with bilateral chronic left greater than right shoulder, neck, and upper back pain, occipital headaches, and hand weakness. She endorsed only mild relief after the cervical surgeries. On exam, she was also provocative at the quadrilateral space, radial tunnel, and carpal tunnel. Her symptoms did not respond to physical therapy, subacromial injections, pain management, or acupuncture. She underwent left isolated PM tenotomy. Her pain and other neuropathic lesions on that side resolved completely and she then elected to have the contralateral PM released.

Patient 9. The 22 y/o LHD female sustained a traction injury while overhead lifting 2 years prior at work and felt a severe “burn.” Thereafter, she suffered right arm global dysfunction with constant 10/10 pain and allodynia, debilitating hand weakness and coldness, and shoulder abduction of 30 degrees. She did not respond to physical therapy, pain management, chiropractors, or assessment by shoulder surgeons. She had been diagnosed with thoracic outlet, complex regional pain syndrome, fibromyalgia, and pain seeking behavior. EMG/NCS was normal. MRI showed a type II SLAP tear and mild insertional supraspinatus tendinopathy, both deemed non-contributory. Her pain was so severe she was considering arm amputation. She underwent isolated PM tenotomy. Her pain significantly improved, with full shoulder ROM restored, and her hand function normalized.

Patient 10. The 25 y/o RHD female administrative assistant presented with severe left chronic shoulder pain, trapezius myalgia, and hand weakness and numbness, diagnosed with WRMD. Over 3 years of occupational health with ergonomics and postural conditioning produced only mild relief. Shoulder MRI was normal. On exam, she was also provocative at the suprascapular notch, quadrilateral space, and radial and carpal tunnels. After isolated PM tenotomy, her pain, myalgia, weakness, and numbness resolved, and she continued to work without issue.

## 4. Discussion

**Figure 5:**
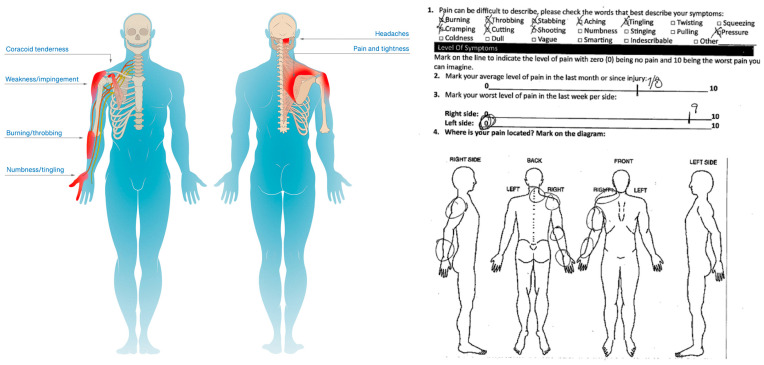
Illustrative Pain Diagram of the Human Disharmony Loop. Constellation of symptoms includes shoulder/neck/upper back pain and tightness; coracoid tenderness; forearm burning, hand numbness and tingling; and pain with overhead reach. Pain is exhibited in C4–6 dermatomes, indicating stretch on these roots from the scapula malposition.

**Figure 6:**
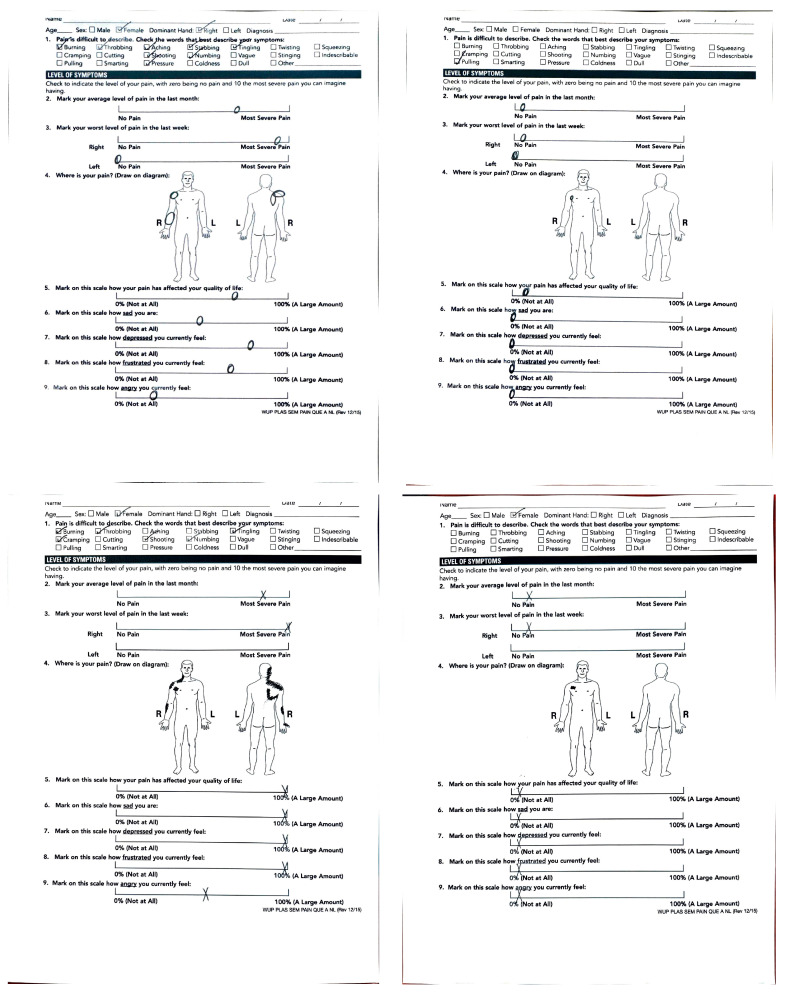
Global Symptom Resolution Following Pectoralis Minor Tenotomy. Illustrative pain diagrams showing resolution of global symptoms after surgery. Top row: Pre-op to post-op for first patient. Bottom row: Pre-op to post-op for second patient. Symptoms are broad but nonetheless tend to categorize as: coracoid tenderness, peri-scapular pain, and C4–6 neuropathy.

In this paper, we present a series of ten challenging patients who originated from diverse etiologies but displayed remarkably similar presentations. All endorsed shoulder pain extending to the neck and upper back, and on physical exam exhibited medial coracoid tenderness, with specific scapula malposition of protraction with anterior tilt and internal rotation (Figure 5). Additional variable symptoms included shoulder weakness; numbness/tingling and burning of the shoulder, forearm and hand; and occipital headaches (Table 2). Moreover, prior treatments failed to help. However, isolated PM tenotomy dramatically improved these patients (Figure 6), which suggests that tightness of this muscle inserting onto the coracoid process of the scapula played a central and unique role in their intractable pathophysiology.

**Figure 7:**
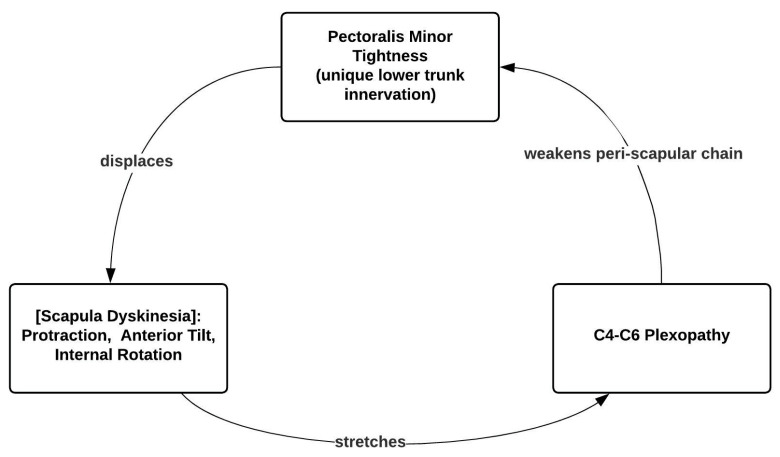
The Human Disharmony Loop. The central positive feedback loop is visualized: pectoralis minor tightness protracts the scapula, which stretches the C4–6 roots, which reinforces pectoralis minor tightness due to its unique lower trunk C8–T1 innervation. The positive feedback nature of the loop produces the intractable and resistant symptomology of chronic pain, neuropathy, and weakness of the human upper limb. Pectoralis minor tenotomy breaks the cycle, reharmonizing the upper limb and obliterating the pathologic sequelae.

From these considerations, we propose a novel clinical syndrome: the Human Disharmony Loop (Figure 7). Our syndrome centers on a positive feedback cycle: PM tightness displaces the scapula, which stretches the upper C4–6 roots, which weakens the remaining peri-scapular chain. Hence, PM tightness reinforces itself. The peri-scapular muscles responsible for coordinating scapulothoracic motion are all innervated by the upper C4–6 roots [2], except for the PM, which additionally receives lower C8-T1 input via the medial pectoral nerve [3]. Anatomically, the upper roots remain far more susceptible to stretch and, therefore, injury than the lower roots [3,4,5]. Hence, any traction to the brachial plexus will preferentially strengthen the PM and weaken the other peri-scapular stabilizers.

As the PM overpowers these other muscles, the scapula displaces to a disequilibrium of protraction, anterior tilt, and internal rotation. Since the scapula dynamically links the thorax to the arm, this pathologizes the entire anatomy of the upper limb girdle (Figure 5). Consequently, the symptoms span from the neck to the fingertips. The shoulder assumes a dropped and internally rotated (“hunched”) posture (Figure 4). The costoclavicular space narrows, producing thoracic outlet syndrome. The subacromial space narrows, impinging the rotator cuff [6,7] (Figure 2). Traction on the C4-6 roots weakens the peri-scapular chain, yielding neuropathic pain in their respective dermatomes, and generates secondary neuropathy at the suprascapular notch, quadrilateral space, and radial and carpal tunnels. The peri-scapular stabilizers further weaken as they fall out of optimal length–tension relationships. Upper trapezius stretch from scapula displacement irritates the occipital nerves to the scalp, producing headaches/migraines. In summary, patients endorse neck, shoulder, and upper back pain and tightness; weakness with overhead reach; limited ROM; occipital headaches; and numbness/tingling and burning of the shoulder, forearm, or hand (Figure 5). Broadly, the constellation of symptoms categorically aligns with the three components of the loop: coracoid tenderness (from PM tightness), peri-scapular pain with rotator cuff impingement (from scapula malposition), and C4–6 neuropathy (from root stretch) (Table 2).

The Human Disharmony Loop is a clinical diagnosis derived from two anatomic criteria and one symptomatic criterion; it is not a diagnosis of exclusion. Crucially, patients must exhibit both key mechanistic determinants: coracoid tenderness and scapula dyskinesia, either static (at rest) or dynamic (with overhead reach). In addition, they must exhibit one of the groups of resultant symptoms (Table 3). Diagnostic testing may be helpful but may be normal or incidental. MRIs may show incidental findings of loop sequelae (i.e., rotator cuff tendinopathy). EMG/NCS may be negative, except for revealing concomitant distal neuropathy, or if the initial inciting entry into the loop is neurologic (i.e., upper trunk block injury). The peri-scapular chain weakness is primarily due to muscle malposition. While all patients must exhibit the two key determinants, they variably display differing degrees of subacromial impingement/weakness, peri-scapular pain, headaches, or secondary neuropathy.

Notably, patients may enter the central loop through either of the three elements. For female athletes, women with macromastia, strong laborers/bodybuilders, or following shoulder trauma or surgery, the PM tightens primarily. For overhead athletes and OSD/WRMD patients, the scapula protracts primarily. For interscalene block injuries, direct plexus traction, radiation after breast cancer, and cervical stenosis, the C4-6 roots are injured primarily. Once inside the loop, patients cycle through the same pathophysiological sequence, explaining the starkly similar presentations (Figure 6). These patients are often historically challenging. They see an array of specialists to treat the separate downstream sequelae of the loop, such as cuff impingement, distal neuropathy, proximal thoracic outlet, upper back tightness, trapezius myalgia, shoulder pain, occipital headaches, etc. However, they respond poorly because the central source—PM tightness—reinforces itself via a positive feedback cycle. Critically, tenotomizing the PM off the scapula breaks this cycle by restoring normal scapulothoracic kinesthetic glide and reharmonizing the anatomy of the upper limb girdle.

The unique lower trunk innervation to the PM deserves mention. We believe this asymmetry may underlie much of the challenging shoulder and arm pathology (Table 4). We hypothesize this innervation results from an evolutionary idiosyncrasy. For our quadrupedal ancestors, coordination between the forepaw and ventral scapular chain is essential for running, leaping, and pouncing. As Homo sapiens are the only obligate bipedal mammal, the human shoulder, which beautifully relies on neurologic and musculoskeletal balance, remains nonetheless highly prone to imbalance because it evolved from a limb optimized for quadrupedal gait. In fact, we suspect all humans are prone to disharmony; notably, chronic neck and shoulder pain plague over 60% of the population [8].

**Figure 8:**
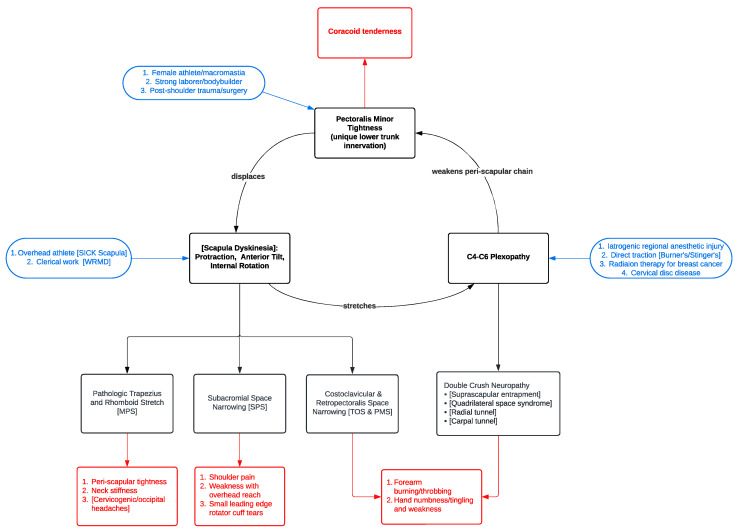
Full Representation of the Human Disharmony Loop. The positive feedback loop in the center is visualized: pectoralis minor tightness displaces the scapula, which stretches the C4–6 roots, which reinforces pectoralis minor tightness due to its unique lower trunk C8–T1 innervation. Diverse groups of patients can enter the loop through different points seen in blue. Clinical signs and symptoms are seen in red. Other challenging and vague syndromes—including scapular dyskinesia, SICK scapula, occupational/work-related musculoskeletal disorder, thoracic outlet, conventional pectoralis minor syndrome, subacromial impingement, cervicogenic/occipital headaches, myofascial pain syndrome, and burners and stingers—are anatomically incorporated into the loop and seen in [brackets]. Pectoralis minor tenotomy breaks the cycle, reharmonizing the upper limb and obliterating the pathologic sequelae.

Our syndrome unifies and elucidates several well-known prior models of upper limb pathology; essentially, we hypothesize that the PM—often overlooked—may constitute the anatomic source behind other challenging and mysterious pathology. Pectoralis minor syndrome is conventionally described as compression of the infraclavicular plexus, producing distal pain and paresthesia [9,10]. However, this alone cannot explain the resolution of proximal thoracic outlet, cuff impingement, scapula malposition, and occipital headaches that we observed. Instead, rather than compressing neurovascular structures akin to other infraclavicular lesions, PM tightness displaces the scapula. TOS remains highly controversial due to the wide variability in presentation, lack of reliable diagnostic testing, and unpredictable response to treatment [3,11]. Both abnormal scapular kinematics and postural derangement are well documented [11]. In our series, we observed obliteration of TOS after PM tenotomy in patients who failed prior targeted anti-TOS therapy. Hence, we suspect some TOS may result from PM tightness, as the scapula displacement narrows the costoclavicular space via the acromioclavicular joint. OSD/WRMD is defined as work- and activity-related pain and tightness of the neck, shoulder, and upper back, with frequent concomitant trapezius and parascapular myalgia, rotator cuff tendinitis, and impingement [1]. Frustratingly, the symptom presentation is vague, the diagnosis is challenging, and the etiology is unknown [1]. But interpreted within the context of our syndrome, these are patients entering the loop via repeated scapular protraction. SICK scapula occurs in throwing athletes and includes asymmetric scapula malposition with coracoid tenderness [12]. However, in this description, the scapula is dyskinetic due to the “ellipsoid shape of the thorax… it tends to ride ‘up and over’ the top … the [PM] and short head of the biceps becomes adaptively tight” [12]. Here, we disagree and reverse the causality: PM tightness pulls the scapula into dyskinesia, which results from an imbalance of muscular forces and not bony shapes. Scapular dyskinesia itself has been observed in TOS, SICK scapula, OSD/WRMD, and throwing and athletic disorders [5,13,14]. Many alleged bony, articular, and neurologic causes have been proposed [14], but whether these are instead effects remains unclear [13]. Crucially, protraction is always observed [14]. We believe our model finally answers the true cause behind the dyskinesia: specifically, the asymmetric neurologic innervation between the ventral PM and the dorsal peri-scapular stabilizers predisposes the scapula to protraction, the vector of pull of the PM (Figure 8).

Our study has important limitations. As a small case series, our results and interpretations must be taken cautiously and replicated or refuted by others. Our volume of PM tenotomy has grown considerably and we intend to refute or validate our hypotheses in future much larger studies; this pilot study is meant to hypothesize a syndrome for others to explore. While our model does account for the similarity in presentation and anatomically explains the clinical improvement observed, there could be numerous unknown confounders in these patients. The diagnosis of PM tightness was based on physical exam, which can be subjective, and future more rigorous studies can implement objective testing as well.

In conclusion, the remarkable motion of the human shoulder mandates a harmony between musculoskeletal and neurologic forces. However, this harmony is prone to imbalance due to the unique lower trunk innervation of the sole ventral chain stabilizer of the scapula, the PM. Disharmony of the system deranges the anatomy of the upper limb girdle and produces a clinical dysfunction that spans from the neck to the fingertips. We propose a novel unifying syndrome that centralizes the PM, causing intractable pain, neuropathy, and weakness in these patients and possibly other poorly understood disorders. Patients presenting with the Human Disharmony Loop may benefit from PM tenotomy. Future research is required to confirm or refute the numerous corollaries of our proposed syndrome.

## 5. Conclusions

The unique asymmetric neurologic innervation to the sole ventral stabilizer of the scapula, the pectoralis minor, renders the human shoulder prone to neurologic and musculoskeletal imbalance. This produces the Human Disharmony Loop: a clinical syndrome spanning from the neck to the fingertips, with chronic pain, neuropathy, and weakness. These challenging and historically intractable patients may benefit from PM tenotomy.

## Figures and Tables

**Table 1:** Patient Characteristics.

Variable	Frequency
Age	37 [26–58] ^1^
Hand Dominance	Right 9 Left 1
Etiology	Breast cancer s/p-radiation 1 Macromastia 1 s/p-reverse TSA 1 Bodybuilder 1 Female athlete 1 Iatrogenic block injury 1 Persistent impingement despite cuff repair 1 Cervical stenosis 1Direct traction (Burner’s/Stinger’s) 1 OSD/WRMD 1
Comorbidities	Diabetes 1 Hypertension 2 Depression 5 Chronic Pain Treated by Pain Management 5 Chronic Pain on Oral Narcotics 4
Pre-Operative Pain	9.0 [7.8–10]
Post-Operative Pain	1.5 [0.8–2]
Pre-Operative ROM ^2^	85 degrees
Post-Operative ROM	180 degrees

^1^ Median [interquartile range]. ^2^ Range of motion (ROM) values of shoulder abduction, for patients with pre-operative limitations.

**Table 2:** Clinical Presentation of the Human Disharmony Loop.

Loop Element	PM Tightness	Scapula Malposition	C4-6 Root Stretch
Symptom/Sign	Coracoid tenderness	Peri-scapular pain and tightness Shoulder weakness and limited ROM (impingement) Occipital headaches	Shoulder and brachium pain and throbbing Forearm burning and throbbing Hand weakness and numbness

Constellation of symptoms and signs aligns with three elements of the loop.

**Table 3:** Clinical Diagnostic Criteria for the Human Disharmony Loop.

Anatomic	Coracoid tenderness + Scapular dyskinesia (protraction either at rest or with overhead reach) +
Symptomatic	3. At least one or more of: Subacromial impingement (shoulder weakness and limited ROM) Peri-scapular pain and tightness (upper trapezius and rhomboid stretch) Neck, shoulder, lateral forearm pain, burning/throbbing Hand numbness/tingling and weakness Cervicogenic/occipital headaches or migraines

Note: Crucially, the Human Disharmony Loop is a syndrome where patients must meet all three strict criteria before being considered for PM tenotomy, as listed here. (“+” means “and”).

**Table 4:** Historically Challenging Patient Populations Who May be in the Human Disharmony Loop.

Chronic shoulder pain Chronic neck and upper back pain and tightness Occipital/cervicogenic headaches Thoracic outlet syndrome (TOS) Occupational shoulder disorder (ORD)/work-related musculoskeletal disorder (WRMD) Subacromial impingement Persistent pain and weakness after shoulder arthroplasty, other shoulder surgeries, or shoulder trauma Persistent distal neuropathy SICK scapula CRPS Fibromyalgia

Note: These historically challenging, intractable patients may be cycling through the loop and should be evaluated for the Human Disharmony Loop signs and symptoms and potential PM tenotomy.

## Data Availability

The original contributions presented in this study are included in the article. Further inquiries can be directed to the corresponding author.

## Abbreviations

The following abbreviations are used in this manuscript:

PM	Pectoralis minor
TOS	Thoracic outlet syndrome
CRPS	Complex regional pain syndrome
OSD	Occupational shoulder disorder
WRMD	Work-related musculoskeletal disorder
ROM	Range of motion
MRC	Medical research council
IRB	Institutional review board
RHD	Right hand dominant
TSA	Total shoulder arthroplasty
SLAP	Superior labrum anterior posterior
EMG	Electromyography
LHD	Left hand dominant
MRI	Magnetic resonance imaging
